# Assessing the reach, effectiveness, adoption, implementation, and maintenance of the ProACTIVE SCI physical activity counseling intervention among physiotherapists and SCI peer coaches during the transition from rehabilitation to community

**DOI:** 10.3389/fneur.2023.1286129

**Published:** 2023-11-22

**Authors:** Kenedy Olsen, Kathleen A. Martin Ginis, Sarah Lawrason, Christopher B. McBride, Kristen Walden, Catherine Le Cornu Levett, Regina Colistro, Tova Plashkes, Andrea Bass, Teri Thorson, Ryan Clarkson, Rod Bitz, Jasmin K. Ma

**Affiliations:** ^1^Faculty of Health and Social Development, School of Health and Exercise Sciences, University of British Columbia, Kelowna, BC, Canada; ^2^International Collaboration on Repair Discoveries (ICORD), Vancouver, BC, Canada; ^3^Centre for Chronic Disease Prevention and Management, Faculty of Medicine, University of British Columbia, Kelowna, BC, Canada; ^4^Division of Physical Medicine and Rehabilitation, Faculty of Medicine, University of British Columbia, Kelowna, BC, Canada; ^5^Spinal Cord Injury British Columbia, Vancouver, BC, Canada; ^6^Praxis Spinal Institute, Vancouver, BC, Canada; ^7^GF Strong Rehabilitation Hospital, Vancouver, BC, Canada; ^8^School of Kinesiology, University of British Columbia, Vancouver, BC, Canada; ^9^Arthritis Research Canada, Vancouver, BC, Canada

**Keywords:** physical activity counseling, spinal cord injury, rehabilitation, physiotherapist, SCI peers

## Abstract

**Introduction:**

Physical Activity (PA) levels for individuals with spinal cord injury (SCI) peak during rehabilitation and sharply decline post-discharge. The ProACTIVE SCI intervention has previously demonstrated very large-sized effects on PA; however, it has not been adapted for use at this critically understudied timepoint. The objective is to evaluate the reach, effectiveness, adoption, implementation, and maintenance of the ProACTIVE SCI intervention delivered by physiotherapists and SCI peer coaches during the transition from rehabilitation to community.

**Methods:**

A single-group, within-subjects, repeated measures design was employed. The implementation intervention consisted of PA counseling training, champion support, prompts and cues, and follow-up training/community of practice sessions. Physiotherapists conducted counseling sessions in hospital, then referred patients to SCI peer coaches to continue counseling for 1-year post-discharge in the community. The RE-AIM Framework was used to guide intervention evaluation.

**Results:**

Reach: 82.3% of patients at the rehabilitation hospital were reached by the intervention. Effectiveness: Interventionists (physiotherapists and SCI peer coaches) perceived that PA counseling was beneficial for patients. Adoption: 100% of eligible interventionists attended at least one training session. Implementation: Interventionists demonstrated high fidelity to the intervention. Intervention strategy highlights included a feasible physiotherapist to SCI peer coach referral process, flexibility in timepoint for intervening, and time efficiency. Maintenance: Ongoing training, PA counseling tracking forms, and the ability to refer to SCI peer coaches at discharge are core components needed to sustain this intervention.

**Discussion:**

The ProACTIVE SCI intervention was successfully adapted for use by physiotherapists and SCI peer coaches during the transition from rehabilitation to community. Findings are important for informing intervention sustainability and scale-up.

## Introduction

Physical activity (PA) levels for individuals who have recently incurred a spinal cord injury (SCI) peak during inpatient rehabilitation and sharply decline after discharge (1). This is unsurprising given the substantial readjustment period experienced by individuals with SCI during the transition from hospital to the community. However, patients are often motivated from seeing their progress during the rehabilitation process, leading to greater interest and commitment to being active post-discharge (2). As such, it has been suggested to promote PA immediately following discharge (2). PA interventions that address the unique needs of people with SCI during the transition between hospital to community are crucial yet understudied.

A limited number of studies have examined the effects of PA interventions at the point of discharge among people with SCI. For example, in the ReSpAct trial, PA counseling was implemented by physiotherapists or sport therapists in 18 rehabilitation centers located throughout the Netherlands (3). Prior to being discharged, patients had an initial PA consultation and subsequently received counseling for 13-weeks post-discharge from trained PA counselors. Patients with diverse disabilities (~2% with SCI) who participated in the program exhibited an increase in their PA and sport participation levels following discharge from rehabilitation. Similarly, an intervention in the Netherlands initiated PA counseling during rehabilitation that was continued by physiotherapists or occupational therapists for 3-months after discharge and demonstrated small to medium-sized effects on PA behavior among those living with subacute SCI (4). These findings are promising; however, clinician time is often limited making the feasibility and scalability of therapist-delivered interventions challenging. There is value in examining other interventionist groups to continue PA counseling post-discharge.

The ProACTIVE SCI intervention is a PA counseling intervention that was co-developed by ~300 end-users including healthcare providers and people with SCI. This intervention has previously demonstrated very large effect sizes for increasing PA behavior that were sustained over 6 months in the research setting (5). The intervention was co-developed with both physiotherapists (community, in-patient, and out-patient) and people with SCI who shared their lived experiences of PA across the rehabilitation continuum. Given the need for PA interventions during the transition from rehabilitation to community, we wanted to explore the adoption of the ProACTIVE SCI when delivered by clinicians and peers across this transition.

SCI peers and health service providers have been identified as preferred messengers of PA information (6). SCI peers can communicate the lived experience of a SCI and help those with a new SCI improve or maintain their PA behavior in the community-setting (7). Further, research has shown peer-delivered PA interventions to be as effective as professionally delivered interventions for increasing PA (8). Physiotherapists have the knowledge and confidence to prescribe exercise and encourage their patients to lead physically active lives (9). To date, no studies have explored the implementation of a coordinated, clinician-to-peer PA counseling service at the point of discharge.

Evaluation frameworks can be used to support implementation through systematic development and evaluation of programs and interventions. A widely used evaluation tool is the RE-AIM framework, which assesses the impact of an intervention across five domains: reach (the percentage of individuals who receive or are affected by a program), effectiveness (the positive and negative consequences of a program), adoption (the proportion of settings of intervention agents that adopt a policy or program), implementation (the extent to which a program is delivered as intended or clients' use of the intervention and implementation strategies), and maintenance (the extent to which a behavior or program becomes routine or maintained over time) (10). This framework was developed to evaluate the impact of public health programs, and now is widely used in many contexts as both a planning and evaluation guide for community interventions at the individual and community level (11). The RE-AIM framework has also been previously used to evaluate PA behavior change interventions (12). Using the RE-AIM framework, the purpose of this study was to evaluate the reach, effectiveness, adoption, implementation, and maintenance of an evidence-based PA intervention for people with SCI delivered by physiotherapists and SCI peer coaches during the transition from rehabilitation to community.

## Materials and methods

### Participants

Interventionists (physiotherapists and SCI peer mentors/SCI peer coaches) were recruited via email from a rehabilitation hospital (GF Strong) and provincial SCI organization (Spinal Cord Injury BC) in Vancouver, BC, Canada. Relevant staff physiotherapists (i.e., those working in the spine or neuromusculoskeletal unit) and SCI peer staff members were contacted by each site's leadership [participant recruitment has been described previously in Ma et al. (5)].

### Study design

This study used a hybrid implementation-effectiveness study design which is the simultaneous testing of an implementation strategy and a clinical intervention (13). A single-group within-subjects, repeated measures design was used. Details of the study design have been previously reported (14). The implementation evaluation is discussed here. The effectiveness (clinical) study evaluates the impact of the ProACTIVE SCI Intervention on patient PA levels and is reported elsewhere (Olsen et al., *in preparation*). Ethics approval for the protocol was granted by the Behavioral Research Ethics Board at the University of British Columbia (H19-02694).

### Implementation intervention

Physiotherapists and SCI peer coaches received an initial, in-person 2-h training (March 2020) on how to deliver the ProACTIVE SCI intervention and were provided with PA counseling forms tailored for each setting. A follow-up two-hour training session for dedicated practice and feedback was conducted 1 month later. It was intended to be conducted in-person, but was conducted via Zoom due to the restrictions of the COVID-19 pandemic. Physiotherapists and SCI peer coaches who attended the initial training session were supported by activities delivered by the champions (dedicated individuals who facilitate implementation) including monitoring, feedback, prompts to continue PA counseling, and problem solving. A prompt was added to physiotherapists' patient-oriented discharge summaries to cue the PA conversation as part of their typical workflow. A third training session (November 2020) was added to re-launch the study after research activities were halted due to the COVID-19 pandemic. Four follow-up training/community of practice sessions were completed over the course of 2 years. See Ma et al. (14) for further details.

### Physical activity counseling and referral procedure

Following the initial training, physiotherapists conducted PA counseling sessions with patients during rehabilitation. Briefly, counseling sessions, grounded in motivational interviewing and the Health Action Process Approach model, involved a discussion to understand client's readiness for PA, goals, barriers, activity preferences, and access to PA resources to then co-develop a PA plan (15, 16). During initial sessions physiotherapists completed a PA counseling form, which was forwarded to SCI peer coaches who would continue counseling sessions with patients for 1-year post-discharge in the community. However, due to the COVID-19 pandemic, counseling in the community did not occur for a 10-month period (March 2020–November 2020). Physiotherapists continued to send PA counseling referrals during this time, but all counseling was delayed in accordance with Public Health recommendations. In November of 2020 the study was re-launched to include the SCI peer coach PA counseling, and the first community counseling session delivered by a SCI peer coach took place in January 2021.

### Measures and analyses

#### RE-AIM

The RE-AIM framework was used to guide the evaluation of the reach, effectiveness, adoption, implementation, and maintenance of the intervention. Descriptions of the RE-AIM elements and how these data were operationalized are described in Table 1.

**Table 1:** Reach, effectiveness, adoption, implementation, and maintenance domains and measurements.

**RE-AIM element**	**Domains as described in Glasgow et al. (10) p. 3–4**	**Measurement of domain**
Reach	The absolute number, proportion, and representativeness of individuals who are willing to participate in a given initiative, intervention, or program	Client discharge summary forms
Effectiveness	The impact of an intervention on important outcomes, including potential negative effects, quality of life, and economic outcomes	Interventionist interviews
Adoption	The absolute number, proportion, and representativeness of: a) settings; and b) intervention agents (people who deliver the program)	Training attendance sheets
Implementation	The intervention agents' fidelity to the various elements of an intervention protocol, including consistency of delivery as intended and the time required. It also includes adaptations made and the costs of the implementation	Interventionist interviews, physical activity counseling forms
Maintenance	The extent to which: (a) behavior is sustained 6 months or more after treatment or intervention; and (b) a program or policy becomes institutionalized or part of the routine organizational practices and policies. Includes proportion and representativeness of settings that continue the intervention and reasons for maintenance, discontinuance, or adaptation	Interventionist interviews, physical activity counseling forms

RE-AIM, reach, effectiveness, adoption, implementation, maintenance; CDS, client discharge summary.

#### Client discharge summaries

Client discharge summaries (CDS) were reviewed by physiotherapist champions to monitor which patients received PA counseling and determine intervention reach. Physiotherapists documented the date the form was completed, if a PA counseling conversation was offered (Yes/No), and reasons as to why PA conversations did not occur (if applicable). The total number of patients who received a full or partial PA conversation (numerator), was divided by the total number of patients with SCI admitted to the rehabilitation hospital during the study duration (denominator) to determine a reach percentage.

#### Semi-structured interviews

Individual semi-structured interviews were conducted over the phone or via Zoom 6-months post training with physiotherapists and SCI peer coaches. Implementation interventions require researchers to strategically combine and borrow from established qualitative approaches to meet the specific needs of the study, which is inherently pragmatic in nature (17). Therefore, a pragmatic qualitative approach was used, in which emphasis is placed on the intersubjectivity of findings (i.e., the idea that there is neither complete objectivity nor subjectivity when interpreting results) (18). Further, a pragmatic approach allowed us to prioritize the translation, co-production of knowledge, and applicability of findings to real world settings (18). Interviews explored factors that affected the effectiveness (i.e., patient benefits), implementation (i.e., intervention timing, feasibility, and impact on interventionist time) and maintenance of the intervention (e.g., program sustainability and scale-up to other rehabilitation centers). Analysis of implementation barriers and facilitators reported in these interviews is reported elsewhere (Lin et al., *in preparation*). Interviews were recorded and transcribed verbatim using Zoom audio transcription software. Zoom-produced transcripts were manually checked by the first author (KO) for accuracy. Interviews were coded using an iterative inductive content analysis approach to map onto the elements within the RE-AIM framework (19). NVivo was used to code transcripts (KO). Codes were then compared by a co-author (JM) to provide feedback and engage in discussion to refine the codes. Member checking was used to confirm and refine the interpretation of the findings.

#### Training attendance sheets

The number of staff who were eligible to participate in the ProACTIVE SCI training was collected using recall from the physiotherapist clinical practice lead (CC-L) at the rehabilitation hospital and the executive director of the provincial SCI peer organization (CM) to determine intervention *adoption*. The number of interventionists attending the training was collected using attendance sheets. Adoption was calculated by taking the number of interventionists who attended the training sessions (numerator), compared to the total number of individuals who were eligible to attend the training sessions (denominator).

#### Physical activity counseling forms

Physiotherapists completed a standardized PA counseling/referral form to document the use of the ProACTIVE SCI intervention components (i.e., discuss current PA levels, goal setting, PA preferences, resources, barriers, and conduct problem-solving and action planning) with patients prior to discharge. SCI peer coaches completed a similar counseling form when conducting PA counseling in the community.

Intervention fidelity (within the *implementation* domain) was assessed by reviewing the use of the initial intake session forms for both physiotherapists and SCI peer coaches. Each section of the form was marked as “yes” if used, and “no” if left blank. Intervention fidelity was calculated by dividing the summed number of form components completed across the forms (as indicated by “yes”) completed by physiotherapists and SCI peer coaches separately, divided by the total number of times these components could have been used. The higher the completion score (0–100%), the greater the fidelity of delivery. Follow-up SCI peer coach PA counseling forms were evaluated as described above but were also summarized for each follow-up session timepoint.

### Sample size

Given the pragmatic nature of the study, we aimed to recruit all eligible physiotherapists (*n* = 13) and SCI peer coaches (*n* = 2). Of note, the SCI peer coaches participating in the study had their regular duties shifted to participate in this role. This limited the recruitment of SCI peer coaches to what was feasible for the organization.

## Results

### Client discharge summaries

Reach percentages were calculated at the patient level (Table 2). One-hundred and forty-one patients were admitted to the rehabilitation hospital with a SCI during the time of recruitment for the study. Of these, 24 patients did not have a PA conversation due to extenuating circumstances, 42 had a partial PA conversation, and 74 of these patients had a full PA conversation with their physiotherapist. Of these 74 conversations, 28 patients consented to participate in the study and receive further PA counseling.

**Table 2:** Client discharge summary completion: intervention reach.

Total # of patients	141	
Total number of patients who did not have PA conversation	25	-
Total number of patients who had partial PA conversation	42	-
Total number of patients who had PA conversation	74	-
Total patient reach (# of patients who had the full PA conversation/total # of patients)	52.48%	-
Adjusted patient reach (# of patients who had partial OR full PA conversation/total # of patients)	82.27%	-
Reason for not having PA conversation	N	%
**Did not have PA conversation**		
Not appropriate due to level of injury	12	17.91%
Missed due to staffing issues	9	13.43%
Patient did not have a SCI	2	2.99%
Patient was moving out of province	1	1.49%
Patient withdrew from therapy	1	1.49%
**PA conversation initiated but terminated before complete**		
Other medical concerns	3	4.48%
Patient was not ready to discuss PA	10	14.93%
No access to phone	1	1.49%
Patient was already very motivated to be physically active on their own (not interested in having conversation)	3	4.48%
Patient declined	24	42.11%
Patient already arranged for private physiotherapy	1	1.49%

CDS, client discharge sheet; PA, physical activity; SCI, spinal cord injury.

### Semi-structured interviews

Analysis of semi-structured interviews resulted in identifying categories within the RE-AIM domains of effectiveness, implementation, and maintenance (Table 3). For effectiveness, both physiotherapists and SCI peer coaches perceived that their patients understood the benefits of PA and receiving PA counseling, as they observed that the information being delivered was effectively used and comprehended by the patients. Four categories were identified related to implementation: (1) physiotherapists found the referral process to the SCI peer organization to be feasible; (2) interventionists identified that there was no single best timepoint for intervening and was instead dependent upon the individual, (3) implementing PA conversations was time efficient for physiotherapists and did not impact their time beyond usual practice; and (4) conducting PA counseling did not change the process of discharge planning for patients, but led to an increase in the use of the SCI PA guidelines by physiotherapists. Three categories were identified within maintenance: (1) resource accessibility and interventionist buy-in are potential barriers to expanding the ProACTIVE SCI intervention to other rehabilitation centers; (2) interventionists anticipated the potential for PA counseling forms to be used among diverse populations beyond SCI; (3) physical activity counseling forms, refresher meetings and ongoing practice sessions are essential implementation intervention components needed to sustain PA counseling delivery.

**Table 3:** Interventionist interview categories and illustrative quotes.

**RE-AIM domain**	**Interview prompt**	**Category**	**Example quotes**
Effectiveness	Do you perceive that your clients are seeing benefits from physical activity counseling (using the Proactive toolkit)? In other words, is it being used or understood? Why or why not?	Patients are understanding the benefits of PA and PA counseling	“*Yeah, I think the majority of clients if the conversation is had you know, in the right way I think they see the value in it. I think a lot of them feel overwhelmed, about going home and having to do this on their own. But I think making that plan and having the conversation is, is a great way to sort of try and overcome that.”* *-Sharon (physiotherapist)*
Implementation	How feasible has it been to refer patients to SCI BC? Should there be a more formal referral process to SCI BC? What should that look like?	Emailing or dropping off a physical copy of the PA counseling form to the SCI peer office on site was perceived as a feasible referral process	“*I think it's been great and should be easy to start. I just scan the form and I can email it through and it's the easiest thing in the world to do. I don't find the conversation that time consuming and I can just have it bit by bit with people. I think it's, it's a very easy process.”* *-Harry (physiotherapist)*
	What are your thoughts on the timing of using the toolkit with your clients (before in-patient discharge or during outpatient physiotherapy)?	Patients have unique needs and optimal times to deliver PA counseling; it's important to at least start the conversation	“*A lot of them are not ready. Lots of them are. You, at least have the conversation, like, do you want to have this conversation. Are you ready for this? And for someone that's just absolutely not. For other ones, it's like, start the conversation, it makes them super anxious and that's not great. And other ones it's like yeah, I'm not really sure but like maybe I would do this.”* -*David (physiotherapist)*
	Apart from training sessions and COP meetings, were there any impacts on your time beyond your usual practice before the implementation?	Implementing PA conversations did not impact time beyond usual practice	“*I would say, it slots in very well into what we do here, you know, it's a quick conversation to have with the client and it's not a large amount of time to put aside for them to do the plan, we would always do a home exercise program anyway on discharge so any, you know, documentation like a, you know, exercise plan sheet with, you know, drawings and instructions of physical activity would always happen anyways I think it just complements the process quite well.”* *-Candice (physiotherapist)*
	Has the use of the toolkit changed your discharge planning and how?	No change to discharge planning, but increased use of the SCI PA guidelines	“*It's changed that about physical activity after discharge. Beyond providing the client with some research in the context of, you know, this is how much physical activity as a minimum and then you can progress to this, having that on the form, you know the 20 min, you know, two times a week of moderate time to high intensity exercise, that particular part of evidence backing up what we are saying is quite helpful.”* *-David (physiotherapist)*
Maintenance	Do you foresee any issues with the uptake of this intervention in other rehab centers?	Resource accessibility and interventionist adoption may be prospective barriers to the expansion of the ProACTIVE SCI intervention to other rehabilitation centers	“*No, just the resource to be the thing. I think just access to resources would be the biggest thing. We have [accessible gym], which is a good resource for us here. I don't think that every rehab center in Canada has access, close access, for clients to an accessible gym.”* *-Julia (physiotherapist)*
			“*You know I'd say the problem would be just the implementation, you just have to a) talk to the physios, and then b) find out if they had buy in. If they had buy in for the importance of it. Then, then kind of, but you wouldn't target that many people right so the problem is if you only have a few spinal cord injury patients that pop up in [Health Authority] once in a while. Having a physical activity conversation will get forgotten.”* -*Cameron (physiotherapist)*
		Potential to use the PA counseling forms with other populations	“*You know I think in other rehab centers it would be nice to not just have you know a SCI physical activity form. That it would just be for all of our clients. You know what I mean. So, I think that there's research, they're looking at this for amputees for example, it would just be nice to have it for across the board because, looking at that form, it could be for my patients with amputations, my patients who have rheumatoid arthritis or any kind of neuromuscular disease transfers, there's so many, you know, I think if you want it to be applied to a variety of rehab centers.”* *-Emily (physiotherapist)*
	If you had the resources to continue sustaining the intervention, what do you feel are the key components that should be kept?	Physical activity counseling form, refresher meetings and ongoing practice sessions are essential implementation intervention components needed to sustain PA counseling delivery	“*I like the idea of having periodic meetings I can't remember what we call them, but we have meetings lunch meetings where [team member] comes in and sort of gives people a little bit of a refresher on the conversation.”* -*Julia (physiotherapist)* “*I think just the practice is helpful like what we'll do on the 24th similar to last time, because yeah, all the things we've done the number one thing with the team liked was actually sitting down and trying to do the coaching.”* -*Rebecca (SCI peer coach)* “*I like the sheet or like just a framework of clients verbalizing what their expectations are and intentions are and what they are planning I think it's a good conversation, and so having the sheets or like a framework to go through is valuable. Just so you have what's in your mind and verbalize it and write it down. So, it's a bit more of kind of really flushing out what someone's plan is afterwards, and they can get a better understanding of it. There is value in that.”* *-Sharon (physiotherapist)*

Pseudonyms were used as place holders for participant names.

### Training attendance sheets

All practicing physiotherapists in the relevant units at the rehabilitation hospital who were eligible for the training attended the initial ProACTIVE SCI training session in March, 2020. Eighty-five percent of the attendees of the first training session attended the second session. Further, 77% of these physiotherapists attended the refresher training in November 2020. All of the designated SCI BC peer coaches received the ProACTIVE training and attended all training sessions.

### Physical activity counseling forms

**Figure 1:**
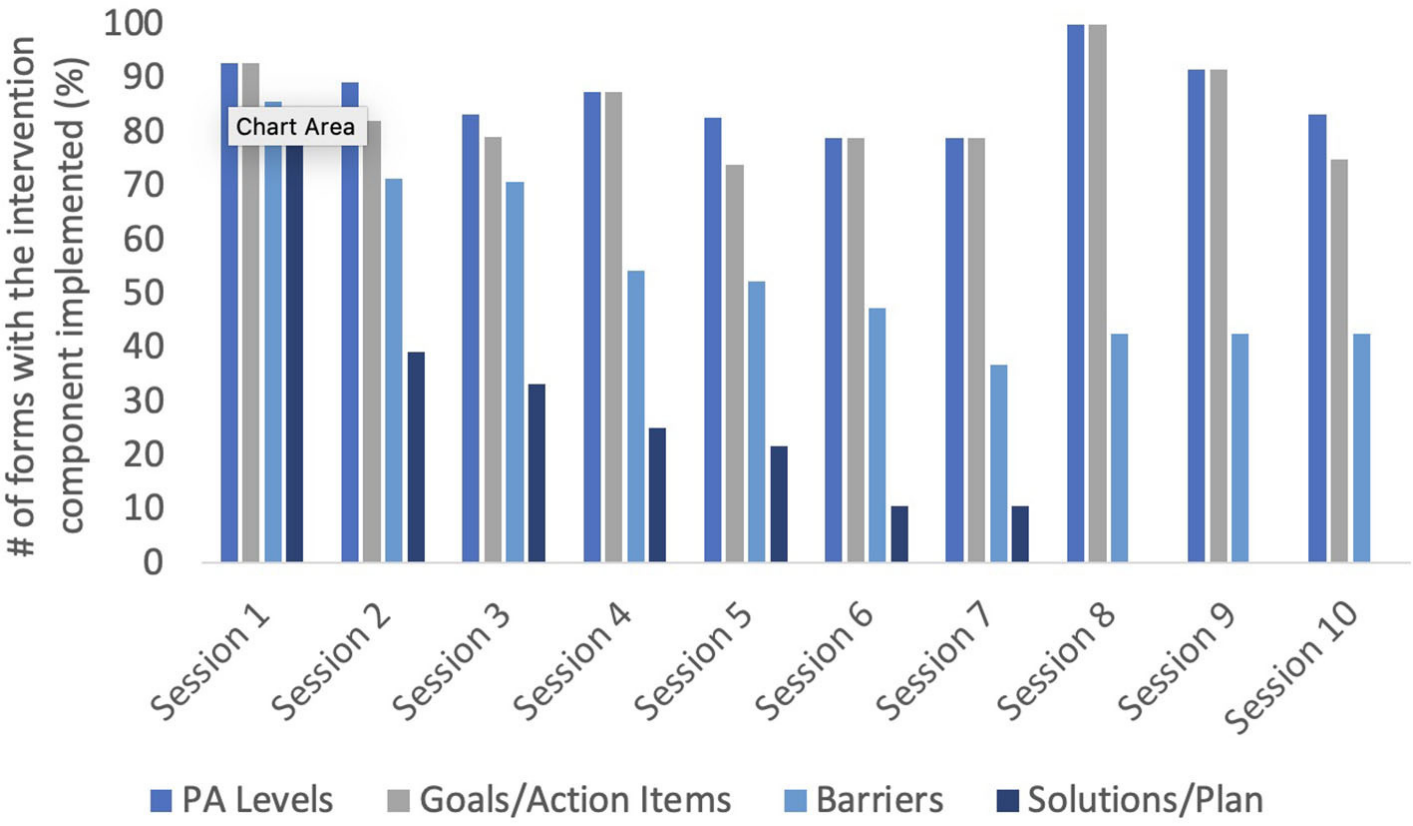
Frequency of PA counseling behaviors delivered by SCI peer coaches during follow-up counseling sessions over time. Not all patients elected to receive all 10 offered sessions (4 patients attended 2 sessions; 1 patient attended 4 sessions; 4 patients attended 5 sessions; 3 patients attended 7 sessions; and 12 patients attended all sessions). Further, 4 patients withdrew from the study after their second counseling session. Therefore, values are reported as percentages relative to the number of patients who attended the session.

Physiotherapists PA referral/counseling forms showed that the most frequently used components of the forms included: whether the patient was interested in discussing PA, goal setting, activity preferences, resources available, potential barriers, and action planning (Table 4). The least used components of these forms were discussing the benefits of PA, current PA levels, and the SCI PA guidelines (Table 4). PA counseling forms, completed by SCI peer coaches, showed that all sections of the forms were used by peer coaches during initial counseling sessions, but the use of these components decreased over time (Figure 1). Specifically, peer coaches consistently discussed patients' PA and goal setting/action planning across all sessions. Discussing barriers to being active was consistently used during sessions 1–5, and slowly decreased in use for the remaining sessions (i.e., sessions 6–10). Lastly, developing a plan/solution for being active dropped substantially in use between the first and second session, and was only occasionally used (i.e., <30% of the time) beyond the third session. Further, this section was not used at all beyond the seventh follow-up session for any patient.

**Table 4:** Physical activity counseling forms: intervention component use frequencies.

**Form component**	**Frequency of component usage by physiotherapists**	**Frequency of component usage by SCI peer coaches during first counseling session**
Are you interested in being more physically active?	100%	95%
What are your goals?	100%	100%
What are you currently doing for physical activity?	30.9%	100%
Benefits of physical activity	8.5%	N/A
SCI physical activity guidelines	0%	N/A
What types of activity do you enjoy/are you interested in doing?	91.5%	88%
What resources do you have available to you?	95.8%	96%
Things that could get in the way (barriers) of your goals?	90.1%	100%
Your plan/timetable	81.7%	90%

A total of 71 PA counseling forms were completed by the Physiotherapists. A total 28 PA counseling forms were completed by SCI peer coaches during their initial exercise counseling sessions with patients. N/A, not applicable.

## Discussion

This study aimed to evaluate the reach, effectiveness, adoption, implementation, and maintenance of an evidence-based PA intervention for people with SCI that was delivered by physiotherapists and SCI peer coaches during the transition from rehabilitation to community. Results showed that the ProACTIVE SCI intervention reached the majority of patients who were admitted to the rehabilitation hospital, suggesting successful adaptation for use during this transitional period. Implementation was supported by high fidelity to the PA counseling intervention components and interview findings suggest the feasibility of the intervention with respect to minimal impacts on time beyond usual practice or discharge planning. Further, the coordination of program delivery between physiotherapists and SCI peer coaches was deemed feasible. This coordinated referral approach along with core intervention components such as ongoing training with opportunities for practice and a PA counseling form is suggested to be integral to the long-term maintenance of the ProACTIVE SCI intervention. While these finding are context-specific, we suggest strategies for future sites to adopt the ProACTIVE SCI at this critically understudied timepoint. More broadly, findings can support the integration of a clinician to peer mentor/coach referral system that links clients from rehabilitation to community in rehabilitation institutes across Canada.

### Reach

This intervention reached 82% of patients admitted to the participating rehabilitation hospital. This is in line with previous research examining the delivery of PA counseling interventions to the general population who were using primary care clinics, which found that 77% of patients were reached by the program (12). The high level of reach in the current study could be due to the ease with which patients could receive this program. PA counseling was integrated into the standard of care patients would receive from their physiotherapist by adding a prompt to their existing patient-oriented discharge summaries. Semi-structured interviews with physiotherapists highlighted that integrating PA counseling conversations was time efficient and naturally fit into the scope of their practice, further supporting the high level of patient reach. However, the initial reach of the program did not directly translate to participating in continued PA counseling in the community program. Specifically, less than half of the patients who received a PA counseling session from their physiotherapist consented to participate in the full intervention (i.e., continue the PA counseling in the community). As mentioned, the time following discharge can be an overwhelming readjustment leading to decreases in PA (1). PA often does not take priority after rehabilitation with competing concerns like housing, accessibility, family, and financial considerations (20). Further, individuals with SCI often face barriers like lack of time, energy, and motivation to be physically active post-injury, making it especially challenging to participate in PA (21). While the intervention reach to patients was high in-hospital, it is possible that not all patients are ready to prioritize PA during the transition to community and further examination is warranted to better support this transition (6).

### Effectiveness

Interviews revealed that both physiotherapists and SCI peer coaches held positive attitudes toward the intervention and highlighted positive perceived benefits for patients participating in PA counseling, including increased planning for PA and increased awareness for managing PA barriers. Theories of behavior change have previously supported the link between attitudes and intention formation, and later behavioral enactment (e.g., Theory of Planned Behavior and the Health Action Process Approach) (22, 23). Interventionists held positive attitudes toward the intervention, which may have contributed to the improved counseling behaviors over time (Shu et al., *under review*). The importance of attitudes in predicting counseling behaviors has been previously supported. Results of a study that investigated the voluntary delivery of HIV counseling among schoolteachers using the theory of planned behavior showed that intention to deliver this counseling was significantly predicted by attitudes toward the intervention (24).

### Adoption

The ProACTIVE SCI intervention was successfully adopted by all physiotherapists at the rehabilitation hospital and all eligible SCI peer coaches. We used an integrated knowledge translation (IKT) approach whereby physiotherapists and people with SCI were involved in the development, delivery and analysis of the research. This allowed for evidence from both research and practice contexts to bi-directionally inform intervention decision-making, and is consistent with the IKT Guiding Principles for conducting SCI research in partnership (25). Interventions that are designed and implemented together with stakeholders are often more likely to be adopted within existing delivery systems (26). For example, an effectiveness-implementation trial compared the adoption of physical activity programs that were iteratively and interactively developed with stakeholders to programs that were unidirectionally disseminated (26). Comparison of the two program design types demonstrated that the two designs showed similar effects on PA behavior, however, programs that involved end-users in development reported significantly greater adoption rates, intentions to sustain program delivery, and participant reach. Organizations wanting to increase adoption rates and potentially support implementation sustainability should seek to meaningfully engage delivering partners as best practice when designing and implementing programs.

### Implementation

When designing clinical interventions, interventionists need to be able to implement these programs into their standard-of-care practice. In the current study, PA counseling was incorporated into typical patient discharge planning, which resultantly had minimal impact on time beyond usual workload. Incorporating non-treatment PA advice during normal consultations has previously shown to be perceived as more feasible than creating a separate PA counseling session (9).

Beyond incorporating the PA counseling conversation into their regular discharge planning, use of PA counseling forms and referral to SCI peer coaches to continue PA counseling post-discharge were critical aspects of the successful intervention implementation. As highlighted in the interviews, physiotherapists found the structure provided by the counseling forms to be helpful in providing a framework when delivering PA counseling (supported by the high fidelity to the core components outlined in the forms), but also offered flexibility to tailor their delivery to the individual. Transferability of interventions to new contexts is often uncertain, and interventions need to be adapted to be successful and effective (27). A systematic review of the adaptation of programs implemented in community settings found the most common intervention adaptations were adding or removing elements to tailor the program to each individual (28). Similarly, SCI peer coaches delivered PA counseling in the community with high fidelity. However, follow-up counseling sessions often employed fewer components of these forms over time as needed. For example, discussing barriers to PA and providing solutions to these barriers was used less frequently in the later counseling sessions. This is unsurprising, as over time, patients likely needed less problem solving as barriers typically are addressed in the earlier counseling sessions. These findings are in line with a previous examination of the behavior change techniques employed using the ProACTIVE intervention in the research setting, where the time spent delivering behavior change techniques related to goals and planning decreased in the follow-up sessions as compared to the initial session (29).

### Maintenance

Interventionists perceived the intervention could be used in other sites as well as among other populations with disability. However, they noted the primary barriers to scaling this intervention to other centers included access to facilities designed to promote accessible PA and interventionist buy-in. Options for PA may be unusable by some clients with SCI due to lack of transportation, building/facility access, inclusiveness amongst programs, or knowledge of staff, as examples (30, 31). With respect to interventionist buy-in, interventionists voiced that maintaining PA counseling as a priority is challenging, though ongoing trainings may help address this challenge. Professionals who are committed to ongoing learning and training have previously been shown to be more effective teachers, and those resistant to receiving training show low levels of program implementation (32). Lastly, while not examined in this study, interventionists foresaw the opportunity for the ProACTIVE SCI intervention to be adapted for use among other populations. Use of physical activity counseling in clinical settings is well-studied among other populations (33–35). However, examination of coordinated referral to peer services from rehabilitation is limited and warrants further study in other clinical populations.

## Strengths and limitations

To our knowledge, this is the first study to examine coordinated PA counseling between physiotherapists and SCI peer coaches at the point of hospital discharge. In Canada, each province has an equivalent SCI advocacy organization. These findings may support the case for modeling this coordinated referral process across the country. Another strength of this work is the integrated knowledge translation approach. Representatives from all involved parties were involved in decision-making from the point of research question inception through to implementation. The importance of this collaborative approach is highlighted by the sustained use of this intervention beyond the project lifecycle as both the rehabilitation hospital and provincial SCI organization have adopted this intervention into standard practice.

As limitations, due to restrictions during the COVID-19 pandemic, any new staff hired onto the spine unit at the rehabilitation hospital were not trained to deliver the ProACTIVE SCI intervention. This may have led to fewer patients having PA counseling conversations and subsequently being referred to SCI peer counseling. Despite a break in the study, the majority of patients did receive PA counseling as reflected in the high reach levels observed in this study. Another limitation was in interpreting the fidelity to the intervention by examining use of the PA forms. Analysis of these forms revealed infrequent discussion of the benefits of PA or the SCI PA guidelines. As these sections were summarized in a diagram, there was no text required to record whether these sections were discussed within the referral form. Other form components (e.g., goal setting) require a text entry from the provider. Therefore these sections may have been delivered to patients, but were undocumented. Lastly, reach data was collected by champions who would periodically review client discharge summaries with the group of physiotherapists to report whether they delivered the ProACTIVE SCI intervention with each patient. It is possible that social pressures may have influenced the reporting or even acted as an intervention component (e.g., social influences) itself. Lastly, the intervention was delivered by only two SCI peer coaches. Since completing the study, additional SCI peer coaches have been trained and it would be valuable to examine the implementation and effectiveness of this interventions amongst a greater diversity of SCI peer coaches.

In conclusion, this study demonstrated that the ProACTIVE SCI Toolkit can be adapted for use by physiotherapists and SCI peer coaches during the transition from rehabilitation to community, a critical and understudied timepoint for PA intervention. Findings are important for informing intervention sustainability and scale-up to other institutions and interventions. Future studies should continue to monitor program maintenance of the ProACTIVE SCI intervention beyond the 6-month period.

## Data availability statement

The raw data supporting the conclusions of this article will be made available by the authors, without undue reservation.

## Ethics statement

The studies involving humans were approved by Behavioral Research Ethics Board at the University of British Columbia (H19-02694). The studies were conducted in accordance with the local legislation and institutional requirements. The participants provided their written informed consent to participate in this study.

## Author contributions

KO: Writing—original draft, Conceptualization, Formal analysis, Writing—review & editing. KM: Conceptualization, Writing—review & editing, Funding acquisition, Methodology, Supervision. SL: Writing—review & editing, Formal analysis, Methodology. CM: Writing—review & editing, Conceptualization, Funding acquisition. KW: Writing—review & editing, Conceptualization, Funding acquisition, Project administration. CL: Writing—review & editing, Conceptualization, Funding acquisition, Methodology, Resources. RCo: Writing—review & editing, Conceptualization. TP: Writing—review & editing, Conceptualization. AB: Writing—review & editing, Conceptualization. TT: Writing—review & editing, Conceptualization, Project administration. RCl: Writing—review & editing, Conceptualization, Project administration. RB: Writing—review & editing, Conceptualization, Project administration. JM: Writing—review & editing, Conceptualization, Formal analysis, Funding acquisition, Methodology, Project administration, Supervision.
